# The role of blinatumomab in adult acute B lymphoblastic leukaemia

**DOI:** 10.1111/bjh.20134

**Published:** 2025-05-14

**Authors:** Anne C. Wilke, Nicola Gökbuget

**Affiliations:** ^1^ Department of Medicine II, Department for Hematology/Oncology Goethe University Frankfurt am Main Germany

**Keywords:** acute lymphoblastic leukaemia, antibody therapy, blinatumomab, monoclonal antibodies

## Abstract

Blinatumomab is a CD19 × CD3‐directed bispecific T‐cell engager that has become an essential backbone of acute B lymphoblastic leukaemia (BCP‐ALL) treatment. It is used in relapsed/refractory disease, minimal residual disease (MRD), as well as in first‐line treatment. Blinatumomab is particularly effective in MRD‐positive BCP‐ALL, inducing a high rate of deep molecular response, with very good overall tolerability. Very promising data are reported for the addition of blinatumomab to first‐line therapy. This review focusses on new aspects of blinatumomab treatment in Philadelphia‐negative BCP‐ALL treatment in adult patients, including MRD‐positive and relapsed/refractory disease, as well as reviewing recent clinical trials implementing blinatumomab into first‐line therapy of BCP‐ALL treatment.

## Graphical abstract

Blinatumomab, a CD19‐directed bispecific T‐cell engager, has become a crucial part of BCP‐ALL therapy in r/r disease, MRD‐positive disease and has recently been implemented into first‐line therapy of BCP‐ALL. This review discusses important clinical trials with a focus on blinatumomab in first‐line therapy of adult BCP‐ALL. *Trials include inotuzumab ozogamicin therapy. 1L, first line; A, adriamycin; B, blinatumomab; BCP‐ALL, B‐cell precursor acute lymphoblastic leukaemia; C, cyclophosphamide; D, dexamethasone; MRD, minimal/measurable residual disease; r/r, relapsed/refractory; V, vincristine.

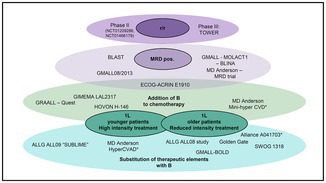

**Abbreviations**
1L: first line
B: blinatumomab
BCP‐ALL: acute B lymphoblastic leukaemia
CAR: chimeric antigen receptor
CNS: central nervous system
CR: complete remission rate with haematological recovery
CRh: complete remission rate without haematological recovery
CRI: Complete Remission with Incomplete Count Recovery
CRS: cytokine release syndrome
CAVD: chemotherapy regime combining cyclophosphamide, vincristine sulfate, doxorubicin hydrochloride, methotrexate, cytarabine and dexamethasone
DFS: disease‐free survival
EFS: event‐free survival
ELN: European Leukemia Net
G‐CSF: granulocyte colony‐stimulating factor
HR: hazard ratio
HSCT: allogeneic haematopoietic stem cell transplantation
InO: inotuzumab ozogamicin
ITT: Intention‐to‐treat population
IV: intravenous
mCR: molecular complete remission
mini‐hyper‐CVD: chemotherapy regime combining cyclophosphamide, dexamethasone, vincristine, methotrexate and cytarabine
MolFail: molecular failure
MolRel: molecular relapse
MRD: minimal/measurable residual disease
NCCN: National Comprehensive Cancer Network
NGS: Next Generation Sequencing
NR: not reached
OS: overall survival
PCR: polymerase chain reaction
PFS: progression‐free survival
Ph negative/Ph positive: Philadelphia‐chromosome negative/positive
POMP: maintenance chemotherapy including 6‐Mercaptopurine, Vincristine, Methotrexate and Prednisone
r/r: relapsed/refractory
RFS: relapse‐free survival
SC: subcutaneous
SoC: standard‐of‐care

## INTRODUCTION

Blinatumomab is a bispecific, directed T‐cell engaging antibody (BiTE) targeting CD19, a pan‐B‐cell marker, and CD3, a part of the T‐cell receptor signaling complex. Binding of CD3 results in recruitment of cytotoxic T cells that are brought in close proximity to CD19‐expressing neoplasms, enabling an effective lysis of malignant cells.^1^ For a detailed mechanistic review, the authors refer to earlier review articles.^2^, ^3^, ^4^ Blinatumomab has proven to be efficacious in B‐cell lymphoma^5^ and B‐cell precursor acute lymphoblastic leukaemia (BCP‐ALL).^6^, ^7^, ^8^ It was initially approved for the treatment of relapsed/refractory (r/r) BCP‐ALL; further trials have shown efficacy in measurable/minimal residual disease (MRD)‐positive disease.^9^, ^10^, ^11^, ^12^ Whereas the treatment of MRD‐positive BCP‐ALL is already part of first‐line treatment, further trials were conducted in order to integrate blinatumomab into the therapy of de novo BCP‐ALL. Blinatumomab also shows efficacy for the treatment of paediatric BCP‐ALL in relapse, as well as Philadelphia (Ph)‐positive BCP‐ALL in adults. This review will focus on the role of blinatumomab in first‐, second‐ and later lines of Ph‐negative BCP‐ALL therapy in adult patients. Clinical trial data will be discussed in chronological order, starting with r/r BCP‐ALL (section ‘Relapsed/refractory B‐acute lymphoblastic leukaemia’), followed by the use of blinatumomab in MRD‐positive disease (section ‘MRD‐positive B‐acute lymphoblastic leukaemia’) and lastly discussing the most recently approved use of blinatumomab in first‐line consolidation, thus MRD‐negative disease (section ‘De novo B‐acute lymphoblastic leukaemia/first‐line treatment’). Important practice points are summarized in Table 1.

**TABLE 1:** Practice points.

Practice points: Blinatumomab in relapsed/refractory disease Blinatumomab is a well‐established and efficacious therapy in r/r BCP‐ALL It should be used in earlier salvage, preferably in lower leukaemia burden, and is usually used for a bridging to HSCT Blinatumomab treatment should be considered early on after detection of relapse when blast count is still low or after a prephase therapy, as side effects and efficacy are dependent on tumour burden New routes of application such as subcutaneous application may improve blinatumomab treatment in the future
Practice points: Blinatumomab in MRD‐positive disease Treatment with blinatumomab in an MRD‐positive setting is efficacious and improves OS; it should be used early on after the detection of MRD persistence or MRD recurrence during/after chemotherapy The optimal time point and MRD level for this intervention depend on the underlying protocol In hCR, blinatumomab treatment is associated with better tolerability and less severe side effects; thus, the use of blinatumomab should not be postponed to open relapse Blinatumomab is an effective treatment to bridge to HSCT In MRD setting, blinatumomab is given without dose step Intrathecal prophylaxis and thorough follow‐up for extramedullary relapses are warranted
Practice points: Blinatumomab in a younger, MRD‐negative patient population in high‐intensity first‐line treatment Blinatumomab can safely be added to consolidation chemotherapy of BCP‐ALL patients and improves OS in MRD‐negative patients, especially in younger patient populations Intensity (number of courses) and time point of blinatumomab treatment is very heterogeneous and requires further evaluation Whether chemotherapy can be omitted due to addition of blinatumomab while maintaining low relapse risk is under investigation Integration of blinatumomab in first‐line therapy will potentially effect treatment in relapsed disease; further trials are needed to investigate relapse after blinatumomab treatment in first line
Practice points: Blinatumomab in an older patient population in first‐line treatment Older patients with BCP‐ALL still suffer from a reduced OS; thus, new treatment strategies in first line are needed Integration of blinatumomab in an age‐adjusted chemotherapy regime can improve OS and is proven to be safe for different protocols In frail patients, individual concepts with low‐dose chemotherapy as combination partner may be needed to avoid toxicity and need further evaluation

Abbreviations: BCP‐ALL, acute B lymphoblastic leukaemia; hCR, haematological complete remission; HSCT, allogeneic haematopoietic stem cell transplantation; MRD, minimal/measurable residual disease; OS, overall survival; r/r, relapsed/refractory.

## RELAPSED/REFRACTORY B‐ACUTE LYMPHOBLASTIC LEUKAEMIA

Blinatumomab is approved for r/r CD19‐positive BCP‐ALL and has become part of international guidelines.^13^, ^14^

In this context, efficacy has been proven in several clinical trials, starting with a phase II study (see Table 2).^6^ A dose escalation protocol was used and 36 patients were recruited to determine safe dose levels. Doses from 5 to 30 μg/m^2^ were administered via continuous IV infusion. As a result, a dose step from 5 to 15 μg/m^2^ was established in order to avoid cytokine release syndrome (CRS) in patients with larger leukaemia burden. This initial dose escalation trial was followed by a larger phase II trial, recruiting 189 patients, in which an initial dose of 9 μg/day was used, followed by a dose step after 7 days to 28 μg/day.^7^ This fixed dose replaced the initial dosing schedule which was based on body surface. Blinatumomab was administered as a continuous IV infusion for 4‐week cycles. Both trials showed complete remission rates with (CR) and without haematological recovery (CRh) of 69% and 43% respectively. Median overall survival (OS) was 9.8 and 6.1 months.^6^, ^7^ Forty percent of CR/CRh patients were able to proceed to allogeneic haematopoietic stem cell transplantation (HSCT) in continuous CR after blinatumomab treatment.^7^

**TABLE 2:** Trials in r/r BCP‐ALL.

Publication/trial	NCT identifier	Phase	Regime	Median age (range) (years)	No. of patients	CR/CRh rate	MRD‐negative rate	RFS	OS
Topp et al.^6^	NCT 01209286	II	Dose escalation, 5–30 μg/m^2^/day in 4‐week cycles	32 (18–77)	36	69% (53–84)	88% of responders	7.6 months (4.5–9.5)	9.8 months (8.5–14.9)
Topp et al.^7^	NCT 01466179	II	9 μg/day for 7 days, 28 μg/day thereafter, 4‐week cycles (max. 9 cycles)	39 (18–79)	189	43% (36–50)	82% of responders	5.9 months for responders (4.8–8.3)	6.1 months (4.2–7.5)
Kantarjian et al.^8^ Jabbour et al.^15^ (TOWER trial)	NCT 02013167	III	9 μg/day for 7 days, 28 μg/day thereafter, 4‐week cycles (max. 9 cycles) Randomized vs. SoC	40.8 (18–80)	376 treated 271 in blinatumomab arm	44% (37.9–50.0)	76% of responders	7.3 months (5.8–9.9)	7.7 months (5.6–9.6)

Abbreviations: BCP‐ALL, acute B lymphoblastic leukaemia; MRD, minimal/measurable residual disease; OS, overall survival; RFS, relapse‐free survival; SoC, standard‐of‐care.

Of all responders in these phase‐II cohorts, 88% and 82%, respectively, achieved a molecular response with MRD levels below 10^−4^ as assessed by real‐time quantitative polymerase chain reaction (PCR).^6^, ^7^ MRD response was associated with a significantly longer OS. Of the 259 patients in the two phase II trials, 18% reached a 4‐year OS.^16^ Of those long‐term survivors, 63% were relapse‐free after 36 months. Within the group of long‐term survivors (>36 months), 49% had received an HSCT in continuous CR within two cycles of blinatumomab, compared to 16% in those surviving less than 36 months.^16^ It is our opinion that we highly recommend performing HSCT in patients achieving a CR after relapse therapy with blinatumomab (see sections ‘The role of allogeneic HSCT’ and ‘Blinatumomab resistance’).

Following these phase II trials, the TOWER study, a phase III, randomized trial, evaluated the efficacy of blinatumomab compared to standard‐of‐care (SoC) treatment, that is, polychemotherapy mostly based on high‐dose cytarabine.^8^ Three hundred seventy‐six patients were treated in this study, with 271 in the blinatumomab arm. The administration of blinatumomab was performed as described previously in the large phase II trial.^7^ CR/CRh rates were 44% in the blinatumomab group, compared to 25% in the SoC arm, resulting in a median OS of 7.7 months for blinatumomab treatment versus 4.0 months in the SoC arm.^8^ Further analysis of the trials in r/r BCP‐ALL led to several important conclusions: (a) Blinatumomab was more effective and associated with improved outcome if used in first salvage^15^; (b) blinatumomab was less effective in terms of remission induction when used in higher leukaemia burden, that is, bone marrow blast count above 50% (see Table 1).^7^, ^17^

All trials in r/r BCP‐ALL investigated the treatment of blinatumomab in 4‐week cycles, and this treatment course has been established in all further protocols.

Shortening of blinatumomab therapy cycles, for example, to 14 days has been discussed with the goal to reduce patient burden and costs. Data on T‐cell expansion during the blinatumomab infusion show that the expansion goes on even after 2 weeks of treatment.^18^, ^19^ It is known that the response on day 14 is predictive of overall response to blinatumomab. However, these data are based on 4‐week standard blinatumomab cycles.^20^, ^21^ Further trials will be required in order to answer the question of whether the efficacy would be similar with a shortened infusion time.

More recently, a subcutaneous (SC) formulation of blinatumomab was tested in a phase Ib trial in r/r BCP‐ALL patients with a dose escalation and further dose expansion design.^22^ Dosing was weekly in the first week and thrice weekly in the subsequent weeks. In 27 evaluated patients, safety and efficacy were convincing with a CR/CRh rate of 86%–92%, depending on the applied dose. Importantly, pharmacokinetic trials underlined that through SC application, potentially higher activity levels could be achieved.^22^ Further trials are needed to evaluate the efficacy of this route of application, which may be preferable as it avoids the implantation of an intravenous (IV) catheter. Furthermore, it may be suitable to achieve higher doses, thereby potentially resulting in better activity.

## MRD‐POSITIVE B‐ACUTE LYMPHOBLASTIC LEUKAEMIA

The first trials with blinatumomab in BCP‐ALL were conducted in MRD‐positive patients in first or second CR. Persistence or recurrence of MRD is the most relevant prognostic factor in BCP‐ALL, independent of age, as it is indicative of resistance to standard chemotherapy.^14^, ^23^ MRD‐positive disease is consequently a major indication for HSCT as summarized in the European Leukemia Net (ELN) and National Comprehensive Cancer Network (NCCN) recommendations.^13^, ^14^ However, positive MRD status before HSCT is associated with a high risk of relapse after HSCT and poorer overall survival.^23^, ^24^, ^25^, ^26^ Furthermore, patients with high levels of MRD tend to relapse before HSCT can be realized. Taken together, these considerations make up the rationale for the current standard approach to reduce MRD burden before HSCT through the use of targeted compounds, like blinatumomab, to improve the chances of long‐term survival after subsequent HSCT.^13^, ^14^ To identify these patients, valid MRD measurements with a sensitivity of at least 10^−4^ (i.e. 0.01%) from a reference laboratory are essential. Furthermore, the applied methods for MRD detection should be considered; quantitative PCR, Next Generation Sequencing (NGS)‐based measurement of clonal immunoglobulin receptor rearrangements or detection of leukaemic cells using multicolour flow cytometry (MFC) can be used. These methods pose different problems; PCR may need a longer time to set up, which might be problematic for early MRD detection, whereas MFC can be challenging in regenerating bone marrow. Lastly, the time point of MRD measurement is crucial and is dependent on treatment protocol. To identify chemotherapy resistance, all relevant chemotherapeutic elements should have been administered before MRD measurement.^14^

Reduction of MRD in patients with molecular failure (MolFail) or molecular relapse (MolRel) with immunotherapy, that is, a different mechanism of action, is an effective strategy to improve remission status and outcome. A pilot trial with 20 MRD‐positive patients (>10^−4^) was initiated and yielded promising molecular response rates of 80%, and a haematological relapse‐free survival (RFS) of 61%, after a median follow‐up of 33 months.^27^, ^28^ Subsequently, the BLAST trial, a phase II trial with 116 treated patients, assessed the benefit of blinatumomab therapy in CR with MRD positivity.^9^ MRD positivity was defined as ≥10^−3^ in first or subsequent CR, after a minimum of three courses of polychemotherapy as an inclusion criterion. MRD positivity was mostly determined via real‐time quantitative PCR of clonally rearranged immunoglobulin and, in a few cases, by MFC. Patients had to be in haematological complete remission (hCR) at inclusion, and those with previous central nervous system (CNS) involvement were excluded. Patients received blinatumomab by continuous IV infusion at a dose of 15 μg/m^2^ without a dose step for up to four cycles. MRD response was assessed after cycle 1. Afterwards, patients were able to proceed to HSCT at any time. MRD response was defined as MRD levels below 10^−4^. Median age was 45 years. Of note, 47% had MRD levels ≥10^−2^ and 35% were in second or later remission. After blinatumomab, molecular complete response (mCR), no target amplification with a minimum sensitivity of 10^−4^ was seen in 80% of all evaluated patients.^9^ In a long‐term follow‐up analysis, OS was found to be 36.5 months, with an estimated 5‐year OS of 43% for all patients.^10^ For MRD responders, median OS was not reached after 5 years, whereas non‐responders achieved a median OS of only 14.4 months. Importantly, OS differed depending on the line of treatment, and a benefit was shown for patients treated in first CR, with a median OS of 41.2 months versus 23.1 months for patients treated in later remission. Of note, this difference was not statistically significant in this analysis.^10^ Overall, 76% of the patients received HSCT, and 41% of patients in the HSCT group were cured. Due to various non‐standard HSCT procedures, including mismatch donors and full myeloablative conditioning in older patients, the mortality in CR after HSCT was high in this trial. On the other hand, the vast majority of patients without HSCT relapsed and only 19% remained in remission.^10^ Therefore, for patients without the HSCT option, consolidation and/or maintenance therapy should be considered after the use of blinatumomab in first CR with MRD positivity. Overall, we recommend HSCT in patients with molecular failure/relapse during or after standard therapies after induction of MRD negativity by blinatumomab in eligible patients.

In a European collaboration, the data from the BLAST trial were compared to historical data with comparable MRD detection measures from SoC, and, when adjusted for HSCT rates in both cohorts, a significant advantage for blinatumomab treatment was demonstrated.^29^

In a subsequent phase II trial, the MRD level for inclusion was lowered to 10^−4^. After an amendment, the trial also included patients with non‐quantifiable MRD.^30^ Interim results of 60 evaluated patients from this study showed an mCR of 67%.^30^ OS was dependent on the MRD level at inclusion, confirming that patients with higher MRD levels had an inferior outcome. Furthermore, patients with MolFail (included with MRD persistence after first‐line therapy) had a superior outcome compared to patients with MolRel (OS 71% vs. 54%).^30^ For further interpretation of these results, full publication of the trial is awaited. In another single‐centre phase II trial, blinatumomab was used in MRD‐positive BCP‐ALL with MRD levels above 10^−4^, including 37 patients with both Ph‐positive and Ph‐negative BCP‐ALL.^11^ Blinatumomab was administered at the same dose level of 28 μg/day for up to nine 4‐week cycles. This considerably longer treatment (as opposed to the up to four cycles in the BLAST trial) was intended to provide an alternative consolidation or maintenance for patients ineligible for HSCT. Forty‐nine percent of patients were, in fact, Ph‐positive and received concomitant treatment with tyrosine‐kinase inhibitors (TKI). Seventy percent of all patients achieved a mCR, including 84% of Ph‐negative BCP‐ALL patients and 61% of Ph‐positive patients. Of note, MRD measurement was conducted by flow cytometry in Ph‐negative patients. For all patients, 3‐year OS was 67%, and relapse‐free survival (RFS) was 63%. In this study, higher MRD levels (MRD ≥10^−3^ vs. MRD <10^−3^) and the use of blinatumomab in second or later CR were associated with an impaired RFS and OS.^11^

In our opinion, the question of how many cycles of blinatumomab treatment are ideal cannot be sufficiently answered. Multiple confounding factors can result in a shorter treatment duration, such as HSCT or response failure with consecutive therapies. These circumstances prohibit a direct comparison. Furthermore, we are convinced that long‐term single drug therapy will impose a high risk of resistance.

In the ECOG‐ACRIN E1910 phase III trial, an MRD‐positive cohort was also treated and demonstrated improved results for those randomized to the blinatumomab arm (for details, see Table 3).^12^, ^31^, ^32^

**TABLE 3:** Trials in MRD pos BCP‐ALL.

Publication/trial	NCT identifier	Phase	Setting	Median age (range) (years)	No. of patients	Complete MRD response	RFS	OS
Gökbuget et al.^9^ Gökbuget et al.^10^ (BLAST trial)	NCT 01207388	II	15 μg/day for 4‐week cycle (no dose step)	45 (18–76)	116	80% (71–87)	Median RFS: 23.6 vs. 5.7 months for responders vs. non‐responders All patients: 18.9 months (12.3–35.2)	Median OS: 38.9 vs. 12.5 months for responders vs. non‐responders All patients: 36.5 months (22.0–NR)
Goekbuget et al.^30^ (GMALL—MOLACT1—BLINA)	NCT 03109093	II	28 μg/day for 4‐week cycle (no dose step)	44 (18–83)	64	67%	‐	NR
Jabbour et al.^11^	NCT 02458014	II	28 μg/day for 4‐week cycle (no dose step)	43 (22–84)	37	70%	61 months	NR (est 3‐year OS 67%)
Litzow et al.^31^ Litzow et al.^32^ Litzow et al.^12^ ECOG‐ACRIN E1910	NCT 02003222	III	SoC chemotherapy for 2.5 months, followed by blinatumomab vs. SoC if MRD+	51 (30–70)	488 enrolled 62 MRD+	‐	‐	Median OS: NR vs. 22.4 months HR 0.39 (0.14–1.10)
Goekbuget et al.^33^ GMALL08/2013	NCT 02881086	III	First‐line trial with the option for CD19‐directed targeted therapy in case of Molfail	35 (18–55)	63 Molfail 40 received blinatumomab	55%	‐	1‐year OS (all patients with Molfail) 84% 3‐year OS (all patients with Molfail) 72%

Abbreviations: BCP‐ALL, acute B lymphoblastic leukaemia; HR, hazard ratio; MRD, minimal/measurable residual disease; NR, not reached; OS, overall survival; r/r, relapsed/refractory; RFS, relapse‐free survival; SoC, standard‐of‐care.

In 2018, blinatumomab was approved for the treatment of BCP‐ALL patients with MRD levels ≥10^−3^ (≥0.1%), who were in first or later CR, and it was the first compound to prove efficacious requiring an MRD‐based inclusion criteria and an MRD‐based end‐point.^34^ MRD‐based use of blinatumomab in patients with MolFail or MolRel at levels above 10^−4^ is currently standard practice and part of all guidelines.^13^, ^14^ This concept has also been evaluated in a large‐phase III trial from the GMALL study group. In this trial, more than 90% of eligible patients with MRD persistence above 10^−4^ after two‐phase induction and one consolidation received blinatumomab followed by HSCT.^33^ The 3‐year OS for Ph− BCP‐ALL was 71% in patients with MolFail, which represents a significant improvement compared to previous trials.^33^

## DE NOVO B‐ACUTE LYMPHOBLASTIC LEUKAEMIA/FIRST‐LINE TREATMENT

**FIGURE 1:**
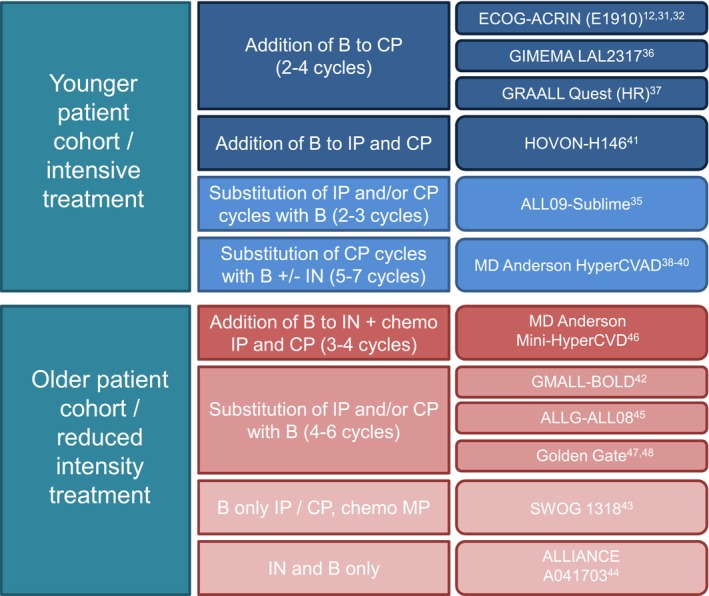
Principles of integration of blinatumomab in 1L therapy of BCP ALL*. An overview of different trials is given depending on treatment strategy (intensive vs. reduced intensity treatment) as well as according to the strategy of integration of blinatumomab (addition of blinatumomab to standard of care therapy vs. substitution of therapeutic elements with blinatumomab). *All protocols include intrathecal prophylaxis. 1L, first line; B, blinatumomab; BCP‐ALL, acute B lymphoblastic leukaemia; CP, consolidation phase chemotherapy; HR, molecularly high risk patients except Ph+; IN, inotuzumab; IP, induction phase chemotherapy; MP, maintenance phase chemotherapy.

Based on results confirming the efficacy of blinatumomab on chemo‐resistant clones in an MolFail setting, a number of trials proposed an integration of blinatumomab in first‐line BCP‐ALL therapy. Blinatumomab was integrated mostly in consolidation,^12^, ^31^, ^32^, ^35^, ^36^, ^37^ while some introduced blinatumomab therapy in an early phase of induction therapy (see Tables 4 and 5).^41^, ^42^ Whereas some trials replaced chemotherapy with blinatumomab,^35^, ^42^, ^45^, ^47^, ^48^ others added blinatumomab to the backbone chemotherapy.^12^, ^31^, ^32^, ^36^, ^37^ and/or integrated blinatumomab along with inotuzumab–ozogamicin (InO).^38^, ^39^, ^40^, ^44^, ^46^ In addition, clinical approaches have varied by age groups, since treatment optimization has different goals for younger and older patients. This section has been divided into high intensive and reduced intensive therapy, and according to therapeutic concept, that is, whether blinatumomab has been added to standard of care (SoC), or whether elements of therapy, that is, chemotherapy blocks, have been substituted with blinatumomab treatment. For an overview, the authors refer to Figure 1 and Tables 4 and 5.

**TABLE 4:** Trials in first‐line BCP ALL—Younger patients/high‐intensity treatment.

Publication/trial	NCT identifier	Phase	Setting	Median age (range) (years)	No. of patients	CR/CRh rate	MRD‐negative rate	RFS	OS
Litzow et al.^31^ Litzow et al.^32^ Litzow et al.^12^ ECOG‐ACRIN E1910	NCT 02003222	III	SoC chemotherapy for 2.5 months, followed by SoC chemotherapy vs. SoC chemotherapy + blinatumomab (4 cycles)	51 (30–70)	488 enrolled 224 MRD neg. MRD− pts. <55 years: 132 MRD− pts. >55 years: 92	81% after SoC induction for entire cohort 75% CR 6% CRi	n.a.	Median RFS: NR (blin + SoC) vs. 71.4 months (SoC); HR 0.46, (0.27–0.78)	Median OS: NR (blin + SoC) vs. 71.4 months (SoC) HR 0.42 (0.24–0.75) 3‐year OS Blin group: 85% SoC group: 68% HR 0.41 (0.23–0.73) MRD− pts <55 years: median OS NR vs. NR HR 0.18 (0.06–0.52) MRD− pts >55 years: Median OS NR vs. 71.4 months HR 0.77 (0.37–1.58)
Short et al.^38^ Jabbour et al.^39^ Short et al.^40^	NCT 02877303	II	Substitution of consolidation chemotherapy cycles with blin ± inotuzumab ozogamicin (5–7 cycles blin)	34 (18–59)	69 38 without inotuzumab ozogamicin 31 with inotuzumab ozogamicin	100% of patients with active disease at study entry	95% of evaluable patients	3‐year PFS 83%	Est. 3‐year OS 87%
Chiaretti et al.^36^ GIMEMA LAL2317	NCT 03367299	II	Addition of 2 blinatomomab consolidation cycles to intensive SoC consolidation	41 (18–65)	149	88%	70% after chemotherapy 93% after blinatumomab	3‐year DFS 66%	3‐year OS 66% (76%, 74% and 49% in the cohorts 18–40, 40–55 and >55 years)
van Baalen et al.^41^ HOVON H‐146	NCT 03541083	II	Addition of blinatumomab to prephase + consolidation to intensive chemotherapy	53 (18–70)	71	58% after prephase incl. blina 95% after 1st blina cons.	46% after prephase incl. blina 91% after 1st blina cons	Est. 4‐year EFS 57% (± 6%)	4‐year OS 76% (±5%)
Greenwood et al. EHA^35^ ALLG ALL09 ‘SUBLIME’		II	Addition of blinatumomab consolidation to a BFM‐based protocol	25 (15–39)	55 (50 evaluable patients)	100%	70.8% (55.9–83.0)	2‐year DFS (ITT) 86.2% (71.5–93.6)	2‐year OS (ITT) 90% (75–96)
Boissel et al.^37^ GRAAL—Quest	NCT 03709719	II	Ph−, molecularly high‐risk patients in CR received blinatumomab consolidation with up to 4 cycles after induction and 1st consolidation accord. to SoC	35 (18–60)	95	n.a.	74%	18‐months DFS 78.8% (66.9–86.8)	18‐months OS 92.1% (83.2–96.4)

Abbreviations: BCP‐ALL, acute B lymphoblastic leukaemia; CR, complete remission rate with haematological recovery; CRh, complete remission rate without haematological recovery; DFS, disease‐free survival; EFS, event‐free survival; MRD, minimal/measurable residual disease; OS, overall survival; RFS, relapse‐free survival; SoC, standard‐of‐care.

**TABLE 5:** Trials in first‐line BCP ALL—Older patients/reduced intensity treatment.

Publication/trial	NCT identifier	Phase	Setting	Median age (range) (years)	No. of patients	CR/CRh rate	MRD‐negative rate	RFS	OS
Goekbuget et al.^42^ GMALL‐BOLD study	NCT 03480438	II	Addition of 4 cycles of blinatumomab to induction/consolidation therapy, omission of 3 chemotherapy cycles	66 (56–76)	50 (47 evaluable for response assessment)	85%	82% of responders	3‐year EFS 60%	3‐year OS 67%
Advani et al.^43^ SWOG Cancer Research Network SWOG 1318	NCT 02143414	II	Blinatumomab induction and consolidation for up to 5 cycles and conventional maintenance therapy for 18 months	75 (66–84)	29	66% (46–82)	Not reported	3‐year DFS 37% (17–57)	3‐year OS 37% (20–55) 2.61 years vs. 1.94 years for <75 years or ≥75 years
Wieduwilt et al. EHA^44^ Alliance A041703	NCT 3739814	II	Induction with up to 2 cycles of inotuzumab ozogamicin, consolidation with up to 3 cycles of blinatumomab	71 (60–84)	33	97%	Not reported	1‐year EFS 75% (61–92)	1‐year OS 84% (72–98).
Fleming et al.^45^ ALLG ALL08 study		II	After induction, consolidation with blinatumomab alternating with B‐cycle of hyper‐CVAD (max. 4 cycles, dose step in first cycle)	51.7 (39.5–66.5)	30	100%	83%	2‐year EFS 60.4%, median EFS 36.1 months	2‐year OS 78.6%, median OS NR
Jabbour et al.^46^	NCT 01371630	II	Mini‐hyper‐CVD and inotuzumab ozogamicin induction, consolidation with POMP and up to 4 cycles of blinatumomab	68 (60–87)	80 (31 treated with blinatumomab)	99% (of all evaluable patients)	94% (best response of all evaluable patients)	5‐year PFS with blinatumomab: 41.8% (28.5–65.0) 5‐year PFS without blinatumomab: 41.8% (17.4–56.1)	5‐year OS with blinatumomab: 40.9% (17.0–63.9) 5‐year OS without blinatumomab: 46.8% (32.5–60.1)
Jabbour et al. ASH^47^, ^48^ Golden Gate Study	NCT 04994717	III	Blinatumomab in combination with low‐intensity chemotherapy vs. SoC	≥55 [reported: 69 (57–77)]	Reported: 14 Recruitment ongoing Planned: 274	92.9% (66.1–99.8) in blinatumomab arm	64.3% in blinatumomab arm	Not yet reported	Not yet reported

Abbreviations: BCP‐ALL, acute B lymphoblastic leukaemia; CR, complete remission rate with haematological recovery; CRh, complete remission rate without haematological recovery; CRi, Complete Remission with Incomplete Count Recovery; DFS, disease‐free survival; EFS, event‐free survival; MRD, minimal/measurable residual disease; OS, overall survival; PFS, progression‐free survival; POMP, maintenance chemotherapy including 6‐Mercaptopurine, Vincristine, Methotrexate and Prednisone; RFS, relapse‐free survival; SoC, standard‐of‐care; ITT, Intention‐to‐treat population.

### Younger patients/high intensity treatment

#### Addition of blinatumomab to SoC therapy

So far, the ECOG‐ACRIN E1910 trial has been the only randomized trial to investigate the addition of blinatumomab to consolidation therapy in MRD‐negative BCP‐ALL.^12^, ^31^, ^32^ This was conducted in newly diagnosed adult patients aged 30–70 years (median age: 51 years), thus evaluating younger as well as older patients. Four hundred and eighty‐eight patients were enrolled and the CR/CRh rate was 81% following induction therapy. Two hundred and twenty‐four patients reached CR and mCR, as assessed by MFC, with a detection level of 10^−4^ after three intensive chemotherapy cycles. These patients were consecutively randomized to receive either four courses of consolidation chemotherapy or to receive up to four courses of blinatumomab, in addition to consolidation chemotherapy (see Figure 1). Following consolidation therapy, maintenance therapy was suggested for 2.5 years. In the blinatumomab arm, OS was significantly improved with a hazard ratio of 0.41 in favour of the blinatumomab arm, and 3‐year OS was 85% in the blinatumomab cohort as opposed to 68% in the SoC cohort.^12^

In our opinion, the rate of randomization, and thus the mCR rate, was quite low, as less than 50% of patients reached mCR and could thus be randomized. Of note, in the control arm of the MRD‐negative patient cohort, 36% of patients died during and after consolidation treatment, mostly due to relapse. It remains to be discussed why the OS was limited in the SoC arm in a population that initially reached MRD negativity.^12^, ^31^

It must be emphasized that, in this trial, the younger patient population (<55 years) particularly seemed to benefit from blinatumomab consolidation, whereas the older patient population had no significant benefit. The reason for this remains unclear.^12^, ^31^, ^32^ The results of the younger patient population have led to revised guidelines, recommending the incorporation of blinatumomab into first‐line therapy in MRD‐negative patients (NCCN) or the consideration of this approach (ELN).^13^, ^14^

The Italian GIMEMA study group conducted a large‐phase II trial that added two cycles (as opposed to four cycles) of blinatumomab to first‐line therapy after two induction cycles and one consolidation.^36^ Of 149 enrolled patients with a median age of 41 (18–65) years, 88% were in CR after induction, and 70% were in mCR after the first consolidation. This proportion was increased to 93% after the first cycle of blinatumomab. Patients with Ph‐like ALL, particularly, showed a high rate of mCR. Three‐year OS and disease‐free survival (DFS) were 71% and 66% respectively. OS differed strongly according to age (see Table 4), and the authors stress the need to improve therapy for older patients. However, in the multivariate analysis, MRD persistence after three cycles of chemotherapy, that is, before the first cycle of blinatumomab, remained the only significant risk factor for an impaired OS. Interestingly, despite a high mCR rate after blinatumomab, the subgroup of Ph‐like BCP‐ALL retained an unfavourable prognosis.^36^ Thus, the blinatumomab‐induced molecular response was apparently not sufficient to improve long‐term outcome. A detailed study report on the realization of HSCT in Ph‐like BCP‐ALL would be of interest. It is currently a matter of debate whether patients with persistent MRD, as frequently observed in Ph‐like ALL, will require subsequent HSCT even if a response to blinatumomab is achieved.

The addition of blinatumomab to SoC treatment was investigated in high‐risk patients (except for Ph+ disease) in the GRAALL‐QUEST study.^37^ This phase II study included 95 Ph‐negative BCP‐ALL patients with molecular high‐risk features, such as KMT2A rearrangement or persistent MRD, and a median age of 35 years. Patients must have reached a continuous CR after induction and first consolidation and were then treated with up to five cycles of blinatumomab, in addition to consolidation and maintenance treatment. HSCT could be performed after the first blinatumomab cycle at the earliest. The 18‐month OS was 92%.^37^ A longer follow‐up is of great interest, especially considering the role of HSCT in this setting.

The trials discussed above included blinatumomab in consolidation therapy. The HOVON study group conducted a phase II trial that included blinatumomab earlier on in the therapeutic algorithm, where a blinatumomab prephase as well as two blinatumomab consolidation cycles were added to a HOVON‐70‐protocol‐based chemotherapy treatment.^41^ Seventy‐one patients with a median age of 53 years (18–70 years), and Ph‐negative and Ph‐positive BCP‐ALL were included. Ph+ disease was treated with a TKI‐based protocol. CR rate in all patients after prephase was 58%, and MRD negativity reported after both blinatumomab prephase and first consolidation cycle with blinatumomab was 46% and 91% respectively. Notably, 15 patients discontinued study treatment before blinatumomab consolidation due to refractory disease, toxicity and early death. Four‐year OS was 76% for all patients, and for Ph‐positive and Ph‐negative patients 85% and 70% respectively.^41^ Particularly the surprisingly good outcome of Ph‐positive patients is to be further evaluated when HSCT rates in this group are reported.

#### Substitution of therapeutic elements with blinatumomab

In the SUBLIME study, blinatumomab was used in place of the second part of induction and reinduction in a Berlin‐Frankfurt‐Münster study group (BFM)‐based protocol for a young patient population (median age: 25 years).^35^ Of 50 evaluated patients, 100% reached CR after blinatumomab treatment, and 71% achieved mCR. The 2‐year RFS of the intention‐to‐treat population (IT was 86%, and 2‐year OS was 90% according to a preliminary analysis.^35^

In an ongoing phase II trial, including 69 patients with newly diagnosed Ph‐negative BCP‐ALL and a median age of 37 years (range: 14–59 years), patients received four cycles of hyper‐chemotherapy regime combining cyclophosphamide, vincristine sulfate, doxorubicin hydrochloride, methotrexate, cytarabine and dexamethasone (CVAD) alternating with high‐dose MTX/AraC followed by a blinatumumab consolidation of up to four cycles. Additionally, four further cycles of blinatumomab were added to POMP maintenance therapy. Starting with patient #39, InO was added to consolidation therapy, and therefore, two blinatumomab cycles were omitted. As one chemotherapy cycle was allowed before study participation, only 53 patients had active disease at study entry. Of these patients, 100% achieved CR. In 59 analysed patients, 95% achieved MRD negativity. The 3‐year continuous remission duration was 83%, and 3‐year OS was 87%.^38^, ^39^, ^40^ In an older population, the addition of blinatumomab after combined InO and low‐dose chemotherapy induction did not improve outcome (see section ‘Older patients/reduced‐intensity treatment’ and Table 5).^46^

Substitution of therapeutic elements with blinatumomab in younger patients is a very interesting approach to possibly improve OS and reduce morbidity. However, further evaluation is needed to verify that other therapeutic elements may be omitted.

### Older patients/reduced‐intensity treatment

Older patients in particular suffer from a reduced OS due to unfavourable disease characteristics and decreased tolerability of age‐adapted paediatric‐based polychemotherapy.^14^ OS was fundamentally improved in a large cohort of older patients (>55 years) by different therapy modulation, such as MRD‐based therapy modification (i.e. availability of targeted therapies in first‐line treatment for MRD‐positive patients) leading to a 3‐year OS of 50%, as opposed to 32%.^49^ This improvement can be attributed to lower induction mortality, significantly higher molecular response rate, and the consequently lower relapse rate due to integration of blinatumomab into the standard backbone. Whereas MRD‐based use of blinatumomab has already become SoC in all age groups, older patients in particular may benefit from the addition of blinatumomab to (reduced) standard chemotherapy, or as a replacement of chemotherapy cycles. Overall, the goal is to increase treatment efficacy and improve treatment tolerability without inducing additional toxicity.

#### Substitution of therapeutic elements with blinatumomab

The GMALL‐BOLD study aimed to sequence age‐adapted polychemotherapy with blinatumomab in induction and consolidation.^42^ Patients aged 56–76 years with newly diagnosed Ph‐negative BCP‐ALL were treated with a shortened first induction phase to reduce leukaemia burden, and the standard second induction phase was replaced by one cycle of blinatumomab. Consolidation was designed to contain three age‐adapted chemotherapy cycles, alternating with three cycles of blinatumomab. Thus, three cycles of standard consolidation therapy were omitted. Maintenance therapy with methotrexate and 6‐mercaptopurine was planned for up to 2 years after initial diagnosis. Median age was 66 years and 40% of all patients had significant comorbidities. Of 50 patients, 76% achieved a CR/CRh, 4% of patients died during induction, which, considering the age and comorbidities of the cohort, is a comparably low number. Forty‐seven patients were evaluated after the first blinatumomab cycle. In this population, CR/CRh was observed in 85%, and of those patients, 82% reached mCR (as opposed to 18% after first induction). Compared to the previous standard treatment, the 3‐year OS was notably improved to 67%, even though this comparison did not reach statistical significance. Consolidation chemotherapy was well tolerated and the mortality in CR was low.^42^

A similar approach was tested by the Australasian Leukaemia and Lymphoma group (ALLG). In a patient group of 40–65 years (intermediate age), blinatumomab was administered alternating with the B cycles of HyperCVAD after a prephase/induction phase.^45^ Of 30 patients enrolled, median age of 51.7 years, 100% achieved CR/CRh and 83% achieved mCR. There were no therapy‐related deaths. The 2‐year OS was 78.6%, and median OS was not reached. Further follow‐up is awaited for this trial.^45^

In the ongoing phase III Golden‐Gate trial, a combination of blinatumomab and low‐intensity chemotherapy is randomized against SoC, which is defined as either a reduced Hyper‐CVAD regimen or the GMALL protocol for older patients.^47^, ^48^ This trial is still recruiting. Preliminary results for a run‐in phase with the blinatumomab arm only show a high response rate and no additional safety signals. However, this run‐in phase was only conducted in 14 patients, so further follow‐up is needed to draw conclusions.^47^, ^48^

Further reduction of chemotherapy was considered by the SWOG (South Western Oncology Group). Patients >65 years were treated with only blinatumomab for induction and consolidation therapy.^43^ They received up to five cycles of blinatumomab followed by a conventional maintenance therapy for 18 months. Median age was 75 years. Of 31 patients, the CR/CRh rate was 66%, and of those, 92% achieved MRD negativity. The limited CR rate underlines the fact that blinatumomab might not be optimal for patients with high tumour burden. Considering the median age of 75 years and possible comorbidities, an alternative approach for induction, for example, mild leukaemia‐reductive chemotherapy with sequential use of blinatumomab, might result in better efficacy. The 3‐year OS was 37% with an age‐dependent reduction of OS. An especially low median OS in the subgroup of patients ≥75 years was observed (see Table 5).^43^ This approach might be feasible for ‘frail’ patients, but the results underline that an approach based on blinatumomab monotherapy does to achieve sufficient efficacy.

To improve efficacy, combination of immunotherapies with different targets may be of interest. The efficacy of InO for the induction of remission in older patients has been demonstrated.^50^ Similarly, the Alliance group carried out a trial in older patients (>60 years) with (up to) two cycles of InO as induction therapy, followed by up to five cycles of blinatumomab consolidation.^44^ In 33 patients with a median age of 71 years, the CR rate was 97%. Although overall, toxicity was within an acceptable realm, one death outside of remission was reported to be due to respiratory failure and sinusoidal occlusion syndrome of the liver. Event‐free survival (EFS) and OS after 1 year was promising (75% and 84% respectively);^44^ however, a longer follow‐up is needed.

#### Addition of blinatumomab to SoC therapy

In a phase II study conducted by Jabbour et al., there was no significant improvement in median RFS or OS after the addition of blinatumomab to a POMP maintenance therapy in an older patient cohort after an induction based on InO and mini‐Hyper‐CVD.^46^ Only 41% of patients completed therapy, mostly due to toxicity or relapse. Forty‐four percent of patients died in remission, mostly due to infection, secondary AML or hepatic sinusoidal obstruction syndrome. These findings underline that combination therapy might be limited due to toxicity and may not always be feasible in an older patient population. It reflects that the choice of dose and sequence of therapeutic elements may be particularly important in an older, frail patient population. Of note, the application of four blinatumomab cycles in a row may be difficult. To interpret and discuss this trial in detail, further follow‐up is needed.

## THE ROLE OF ALLOGENEIC HSCT

Even though there are some cases that can be cured with blinatumomab treatment,^16^ blinatumomab cannot be seen as a substitute for HSCT in patients with MRD‐positive or r/r BCP ALL. These patients should be considered for HSCT if eligible, possibly after a bridging therapy with blinatumomab, and ideally with an MRD‐negative disease status, or, in any case, in the best possible remission after bridging treatment.^23^, ^24^, ^25^, ^26^

In a long‐term follow‐up analysis of two cohorts in r/r BCP‐ALL, 70% of all relapse‐free survivors received HSCT, and 30% of all relapse‐free survivors did not^16^; for further trials with blinatumomab in r/r BCP‐ALL, there is no long‐term follow‐up available to thoroughly address this issue. In the MRD‐positive patient population of the BLAST trial, 41% of patients that were able to proceed to HSCT were cured and remained in long‐term remission, as opposed to 19% of patients who received blinatumomab consolidation only (see section ‘MRD‐positive B‐acute lymphoblastic leukaemia’).^10^ Overall, these trials were not designed to answer the question of whether HSCT should be used as consolidation treatment. Decisions of whether patients proceed to HSCT or are considered ineligible as well as transplantation settings, that is, conditioning regime and donor types may vary and are not comparable between these studies. In conclusion, there are no randomized trials available to address the question of whether HSCT as consolidation after blinatumomab bridging is adequate. However, in a high‐risk cohort, such as r/r BCP‐ALL or MRD‐positive disease, we strongly encourage an HSCT consolidation concept in a younger patient population (up to 55–60 years) according to current guidelines.^13^, ^14^ For older patients, there is no clear consensus and data are sparse. If HSCT is considered for older patients, potential treatment‐related mortality as well as alternative consolidation therapies in those patients who are not eligible for HSCT should be taken into account.^13^, ^14^

## ADVERSE EVENTS

Blinatumomab therapy entails distinct groups of adverse events that are clinically significant in terms of therapy management: (i) neurotoxicity including seizures, (ii) fever and CRS, (iii) cytopenia and infections including infusion line‐related infections and (iv) aberrant laboratory findings. All adverse events are seen more frequently during the first one to two cycles.

### Neurotoxicity

Neurological events often become clinically apparent via signs of encephalopathy, such as dizziness, tremor and aphasia. This can progress to higher grade encephalopathy, including loss of consciousness, if not managed adequately.^7^, ^8^, ^9^ Seizures may occur independently of encephalopathy. To detect neurotoxicity early, patients and caregivers/relatives should be instructed. Regular writing tests should be obtained to identify early signs of encephalopathy; interventions consist of dexamethasone application and infusion interruption. Dose reduction from 28 to 9 μg/day should be discussed at restart in case of severe or repeated neurotoxicity. With this management, neurotoxicity mostly resolves without sequelae. Furthermore, neurotoxicity of higher grades (3/4) is less common compared to other immunological treatments such as chimeric antigen receptor (CAR) T‐cell therapy.^7^, ^8^, ^9^, ^51^, ^52^ Headache may occur frequently, but independently of neurotoxicity, and can usually be managed with paracetamol or metamizol. For further considerations involving the occurrence of neurotoxicity, see Table 6.

**TABLE 6:** Symptoms and management of neurotoxicity.

Clinically significant neurological event	Lead symptoms	General considerations^a^
Encephalopathy type	Tremor Aphasia Dizziness Confused state Depressed level of consciousness Ataxia Memory impairment Dysarthria Apraxia	Administer steroids (e.g. dexamethason at least 3 × 8 mg/day and reduce stepwise) Consider treatment interruption Consider restart at lower dose level after resolution to ≤Gr 1 and after at least 3 days Consider diagnostic work up with imaging (CT/MRI) and CSF analysis Administer supportive care measures as clinically appropriate
Convulsive type	Convulsion Generalized tonic–clonic seizure	Anticonvulsive treatment with consecutive secondary prophylaxis Consider treatment interruption depending on grade Consider restart at lower dose level after resolution to ≤Gr 1 and after at least 3 days Consider diagnostic workup with imaging (CT/MRI) and CSF analysis Administer supportive care measures as clinically appropriate

Abbreviations: CSF, cerebrospinal fluid; CT, computed tomography scan; MRI, magnetic resonance imaging.

^a^ For specific management of neurotoxicity, always consult product information.

### Fever and CRS

CRS is an inflammatory response that usually starts with fever and may aggravate into a life‐threatening vasodilatory shock. In clinical trials with blinatumomab in r/r BCP‐ALL, so in patients with active disease, a CRS rate ≥ grade 3 in up to 4.9% of patients has been reported.^6^, ^7^, ^8^ This risk has been decreased by the dose step schedule.^7^ Relevant CRS is rare in patients in haematological remission (i.e. in an MRD‐directed treatment, or in a consolidation regime in MRD‐negative patients).^9^, ^11^, ^12^, ^39^, ^45^ However, pyrexia, that is, fever, is common at the initiation of treatment and should be addressed with supportive measures such as antipyretics and IV hydration. For reduction of CRS risk and achievement of better efficacy, a debulking prephase treatment should be considered in patients with haematological relapse. Debulking strategies may include prephase treatment with dexamethasone, vincristine and/or cyclophosphamide. InO for debulking is another option, whereas more intensive debulking is not recommended.

Even if CRS ≥ grade 3 occurs, it is mostly manageable by treatment interruption, steroid application (e.g. 3 × 8 mg dexamethasone) and supportive therapy (e.g. hydration, antipyretics, oxygen). It is rarely a cause for permanent treatment discontinuation. If CRS is not responsive to standard therapy, treatment with the IL‐6 inhibitor tocilizumab can be successful.^53^ Fortunately, such an event is rare in blinatumomab treatment.

### Cytopenia and infection

In r/r BCP‐ALL patients, grade 3/4 neutropenia occurs frequently,^8^ often at presentation and is augmented in patients with non‐response. This is mostly due to bone marrow infiltration and not as much attributable to blinatumomab. Thus, the overall incidence for severe neutropenia is 38%, compared to 16% of patients in CR with MRD positivity.^8^, ^9^ Mostly, neutropenia does not require treatment interruption, usually does not lead to relevant infections and can be well handled with short‐term granulocyte‐colony stimulating factor (G‐CSF) application.

However, severe infections were also reported^39^ implying that standard anti‐infective prophylactic treatment, such as anti‐viral prophylaxis with, for example, aciclovir and pneumocystis‐jirovecii pneumonia prophylaxis, should be considered depending on clinical status and concomitant therapy. Furthermore, as hypogammaglobulinaemia was seen upon blinatumomab treatment, immunoglobulins should be monitored and replacement should be considered on an individual basis during blinatumomab therapy.

As central IV lines are a requirement for continuous blinatumomab administration, central IV catheter infections pose an additional risk to patients. However, this risk has not been proven to be significantly higher than with other therapies, that is, chemotherapy.^8^, ^9^ This risk should still be considered and patients and caregivers dealing with continuous blinatumomab infusions at home should be instructed accordingly. A subcutaneous application route for blinatumomab is currently under development.^22^

### Aberrant laboratory values

A common, but less severe side effect is elevation of liver enzymes.^7^, ^9^, ^39^ While generally well manageable and not disruptive to treatment, it is mostly an inflammatory effect due to T‐cell activation.

## BLINATUMOMAB RESISTANCE

Relapse during or after blinatumomab treatment remains a challenging situation. In terms of resistance mechanisms, there are four important considerations for blinatumomab response/relapse: (i) lack or loss of CD19 expression, resulting in a loss of target, (ii) lineage switch, (iii) immune evasion, for example, via exhaustion of T‐cell compartment and upregulated regulatory T cells and (iv) extramedullary relapse.^2^, ^54^, ^55^, ^56^, ^57^, ^58^, ^59^, ^60^, ^61^, ^62^, ^63^, ^64^

In terms of loss of CD19, there are several mechanisms underlying this phenomenon such as frameshift and missense mutations, splicing variants^55^, ^56^ or disrupted membrane trafficking.^57^ Furthermore, switch to myeloid lineage, especially in MLL1/KMT2A‐rearranged disease, may also effectively lead to loss of target.^58^, ^59^ These evasive mechanisms strongly support the use of blinatumomab in combination regimens. The incidence of CD19‐negative relapses is around 20%–30%, thus, less frequent than after CAR‐T‐cell therapy.^58^, ^60^

Immune evasion correlates with limited T‐cell expansion and a higher number of T‐regulatory cells.^61^ PD1/PD‐L1 interaction, as well as T‐cell exhaustion, may be further mechanisms of immune evasion.^62^, ^63^ Phase I/II studies investigating the effect of blinatumomab in combination with checkpoint inhibition in r/r ALL are still ongoing (NCT03160079, NCT03512405, NCT02879695).

BCP‐ALL patients treated with blinatumomab carry a risk of extramedullary relapse depending on the treatment schedule. In one trial, 43% of relapses or treatment failures involved extramedullary sites. Furthermore, a history of extramedullary disease was associated with a lower response rate.^60^ Potential sites of extramedullary relapse are the kidney, CNS, lymphatic and musculoskeletal tissue.^64^

Clearly, the occurrence of extramedullary relapse, including CNS relapse, is a potential cause of failure during/after blinatumomab treatment. The proportion of CNS relapse was as high as 39% in one trial.^60^ Therefore, intensive CNS prophylaxis with intrathecal therapy is recommended during blinatumomab therapy. Although there is the hypothesis that blinatumomab can cross the blood–brain barrier,^65^ there is no sufficient evidence favouring blinatumomab in the setting of CNS or other extramedullary relapse. In rare cases of CNS relapse combined with MRD, we would recommend to clear CSF by intrathecal therapy before potentially using blinatumomab for the management of MRD. Overall, close surveillance for signs of extramedullary relapse is recommended. Extrapolating from this experience and from results in Non‐Hodgkin's lymphoma,^66^, ^67^ where higher doses of blinatumomab (above 100 μg/day) are needed for effective treatment, blinatumomab is not a preferred option to treat extramedullary relapse in BCP‐ALL.

Blinatumomab is an efficacious treatment, particularly in the context of MRD positivity. Due to the possible development of resistant clones, patients with r/r or MRD‐positive BCP‐ALL should be assessed for HSCT. In patients ineligible for HSCT, different consolidation strategies should be considered as blinatumomab monotherapy bears a high risk of relapse. A phase I/II trial investigating the combination of blinatumomab treatment with venetoclax in r/r ALL is currently ongoing (NCT05182385)^68^ and may also offer an interesting approach for patients in later lines and/or ineligible for HSCT.

## ALTERNATIVE TREATMENTS

In terms of treatment alternatives in different situations, alternative targets such as CD22 or different modalities such as antibody‐drug conjugates or CAR T cells may be considered.

InO is an antibody‐drug conjugate with an anti‐CD22 antibody linked to the cytostatic drug calicheamicin. In r/r BCP‐ALL with blasts >5%, InO is an important treatment option, with a reported CR/CRh rate of 68%–81% in different clinical trials, with a median OS of 7.4–7.7 months.^69^, ^70^, ^71^ Median OS is thus comparable to blinatumomab treatment, though in a different patient population. Patients with peripheral blasts above 10 x 10^9/L were not included in the trials, but the bone marrow blast count did not impact response rates, in contrast to blinatumomab. Like blinatumomab, InO was more effective in earlier salvage. There are some data showing efficacy in extramedullary disease.^72^ Data involving the use of InO in an MRD‐positive setting are still sparse, although InO also shows efficacy in MRD‐positive patients.^73^, ^74^ Furthermore, when considering HSCT as consolidation after InO, the increased risk of hepatic veno‐occlusive disease/sinusoidal obstruction syndrome needs to be considered, a severe complication which may be fatal.

CD19‐directed CAR T cells have proven efficacious in BCP‐ALL patients with advanced relapses.^51^, ^52^, ^75^ In children and young adults with r/r BCP‐ALL, Tisagenlecleucel achieved an overall remission rate of 81% (66% in the intent‐to‐treat analysis (ITT)), a median EFS of 24 months and an OS of 63% at 3 years.^51^, ^76^, ^77^ Brexucabtagene autoleucel has been investigated in a population with a median age of 40. Of the treated population, 71% reached CR/CRh. Of note, in only 77% of cases could treatment be administered due to the manufacturing process a limitation that is not a concern with off‐the‐shelf products. OS was 18.2 months but was not reached by the CR population.^52^, ^78^ Obecaptagene autoleucel, a product with reduced toxicity in terms of CRS and immune effector cell‐associated neurotoxicity syndrome (ICANS) has shown similar results in terms of response in a patient population with a median age of 50 years; of all patients that received the product, median overall survival was 15.6 months.^75^ The availability of CAR T‐cell products for adults is still limited in many countries, and these treatments are often allocated to later treatment lines including relapse after HSCT. Clinical trials that identify the ideal introduction of these approaches in earlier treatment lines are warranted.

## CONCLUSIONS AND FUTURE QUESTIONS

Blinatumomab is a highly efficacious treatment for CD19‐positive BCP‐ALL, with great therapeutic benefit for patients with MRD‐positive disease. If possible, it should be used early on in a relapsed/refractory setting, before frank haematological relapse occurs, and can be used to bridge eligible patients to HSCT. In a first‐line setting, there is evidence that blinatumomab can safely be administered, possibly resulting in a reduction of conventional chemotherapy, particularly in older patients, which may result in reduced non‐relapse morbidity and mortality. Furthermore, blinatumomab treatment in MRD‐negative patients in first remission prolonged OS in a randomized phase III trial, which resulted in the integration of first‐line blinatumomab treatment in different international treatment guidelines. However, there are many unanswered questions, including the optimal duration of blinatumomab treatment, the optimal introduction into therapy, as well as the number of cycles. Whether or not the omission of chemotherapy is feasible, especially with respect to potential extramedullary relapse, is also in question. These points ought to be addressed in prospective clinical trials. Additionally, the treatment of relapse after blinatumomab use in first line may become more challenging if resistant clones appear earlier on, possibly reducing the potential benefit of CD19‐directed therapies in r/r BCP‐ALL. Further clinical research and discussion are needed to sufficiently answer these questions as it remains challenging to determine how to best integrate this beneficial treatment in BCP‐ALL therapy. A further concern is how this expensive and challenging therapy can be made available in low‐ or middle‐income countries (LMIC). Off‐the‐shelf products like BiTEs can be of help, as opposed to, for example, CAR T‐cell products. Nonetheless, this may not be entirely feasible as there is still a large discrepancy between therapeutic need and availability in a large part of the world.

## AUTHOR CONTRIBUTIONS

ACW and NG wrote and revised the manuscript together and drafted and edited tables and figures.

## CONFLICT OF INTEREST STATEMENT

ACW has received travel grants from Gilead/Kite, Abbvie and Lilly and speaker honoraria from Roche and Janssen‐Cilag. NG has received institutional research funding from Amgen, Clinigen, Incyte, Jazz, Novartis, Pfizer and Servier and has received speaker honoraria or fees for advisory board participation from Amgen, AstraZeneca, Autolus, Clinigen, Gilead, Incyte, Jazz, Novartis, Pfizer and Servier.
